# Polypharmacy Moderates Link Between Instrumental Daily Activities and Physio-Cognitive Decline in Older Adults

**DOI:** 10.1093/geroni/igaf122.305

**Published:** 2025-12-31

**Authors:** Wan-Yun Chou, Su-I Hou

**Affiliations:** University of Central Florida, Orlando, Florida, United States

## Abstract

**Introduction:**

Physio-cognitive decline syndrome (PCDS) increases the risk of dementia, disability, and mortality in older adults. However, little is known about the role of polypharmacy in the association between instrumental activities of daily living (IADL) and PCDS.

**Methods:**

This cross-sectional study examined the moderating effect of polypharmacy on this relationship among 183 community-dwelling adults aged 65 and older undergoing annual health screenings (March 2021–November 2022) in Taipei, Taiwan. Participants were classified into two groups: robust or PCDS. PCDS was diagnosed if a subject met a cutoff below 1.5 standard deviations of age-, sex-, and education-matched norms of the Chinese version of the Mini-Mental State Examination (CMMSE) and physical (with weakness/ and/or slowness) criteria. PCDS was identified as 21% of participants. Results showed that IADL was negatively associated with PCDS, but this relationship was moderated by polypharmacy. When polypharmacy was present, the association was more potent, and the relationship was stronger, whereas without polypharmacy, the relationship was weaker. These findings highlight the importance of managing polypharmacy alongside daily activity to protect cognitive and physical health in older adults. It is necessary to pay attention to polypharmacy and daily activity among older adults. Targeted interventions addressing both factors may help mitigate PCDS. While the convenience sampling method may limit generalizability, further research is needed to explore the impact of polypharmacy on the relationship between IADL and PCDS identification.

